# Improving hypertension control in Nigeria: early policy implications from the Hypertension Treatment in Nigeria program

**DOI:** 10.1186/s41256-024-00368-9

**Published:** 2024-07-15

**Authors:** Oluwabunmi Ogungbe, Chibuzor Abasilim, Mark D. Huffman, Dike Ojji, Hypertension Treatment in Nigeria Program Team

**Affiliations:** 1 Johns Hopkins School of Nursing, Baltimore, MD USA; 2 Johns Hopkins Bloomberg School of Public Health, Baltimore, MD USA; 3 Division of Environmental and Occupational Health Sciences, School of Public Health, University of Illinois Chicago, Chicago, USA; 4 Department of Medicine and Global Health Center, Washington University in St. Louis, St. Louis, USA; 5 The George Institute for Global Health, University of New South Wales, Sydney, Australia; 6 Department of Internal Medicine, Faculty of Clinical Sciences, University of Abuja, Abuja, Nigeria; 7 Department of Medicine, Cape Heart Institute, University of Cape Town, Cape Town, South Africa

**Keywords:** Hypertension, Nigeria, Health Policy, Delivery of Health Care, Integrated, Implementation Research

## Abstract

Hypertension poses a significant health burden globally. In Nigeria, hypertension prevalence is on the rise, with low rates of awareness, treatment, and control. This policy brief explores the critical gaps addressed by the Hypertension Treatment in Nigeria (HTN) Program, highlighting its strengths, initial outcomes, and scalability in primary care settings. The HTN Program employs an integrated, multilevel care model based on the World Health Organization’s HEARTS technical package, including patient registration and empanelment, team-based care, training and supervision, a standardized treatment protocol, a health information management system, and a drug revolving fund to improve medication accessibility. By December 2023, hypertension treatment and control rates reached surpassing 90% and 50%, respectively, thus underscoring the program’s impact. The HTN Program serves as a model for delivering integrated hypertension care in primary care. Results should be leveraged for political commitment and financing to evaluate and manage non-communicable diseases such as hypertension in primary care through federal and state primary health development agencies. Furthermore, incorporating metrics related to hypertension control and treatment into the Integrated Supportive Management Information System can enhance routine monitoring and evaluation.

## Background

Hypertension is a leading risk factor for cardiovascular and kidney disease globally, contributing to over 10 million deaths each year [1]. In Nigeria, hypertension prevalence has been increasing over the past several decades, with recent estimates ranging from 22% to 44%, which varies by region [2]. However, awareness, treatment, and control rates remain low. In a 2021 meta-analysis, among Nigerians with hypertension, only 29% were aware of their diagnosis, 12% were on treatment, and a mere 3% achieved control [2], whereas other studies have reported modestly higher control rates [3]. These poor control rates substantially increase the risk of complications such as myocardial infarction, strokes, heart failure, and kidney disease.

Currently, key barriers to improving hypertension outcomes in Nigeria include health system challenges like inadequate financing for non-communicable diseases (NCDs), poor integration of NCD care in primary health services, frequent medication stock-outs, limited health insurance coverage, and a limited workforce capacity for hypertension management [4]. From the patient perspective, low health literacy, poverty, inability to afford medications, poor access to care, and challenges with long-term medication adherence remain major bariers. The World Health Organization’s (WHO) HEARTS technical package outlines strategies for health systems to improve care for cardiovascular health in primary healthcare settings [5]. The growing scientific discipline of dissemination and implementation research has been used to identify, adapt, implement, and evaluate effective models for hypertension control in several countries, including in Nigeria. The aim of this policy brief was to describe early policy implications for the wider adoption and scale-up of the Hypertension Treatment in Nigeria (HTN) Program. This policy brief will highlight the program’s strengths, initial outcomes, and potential for scalability within primary care settings.

## Overview of Hypertension Treatment in Nigeria (HTN) Program

**Fig. 1:**
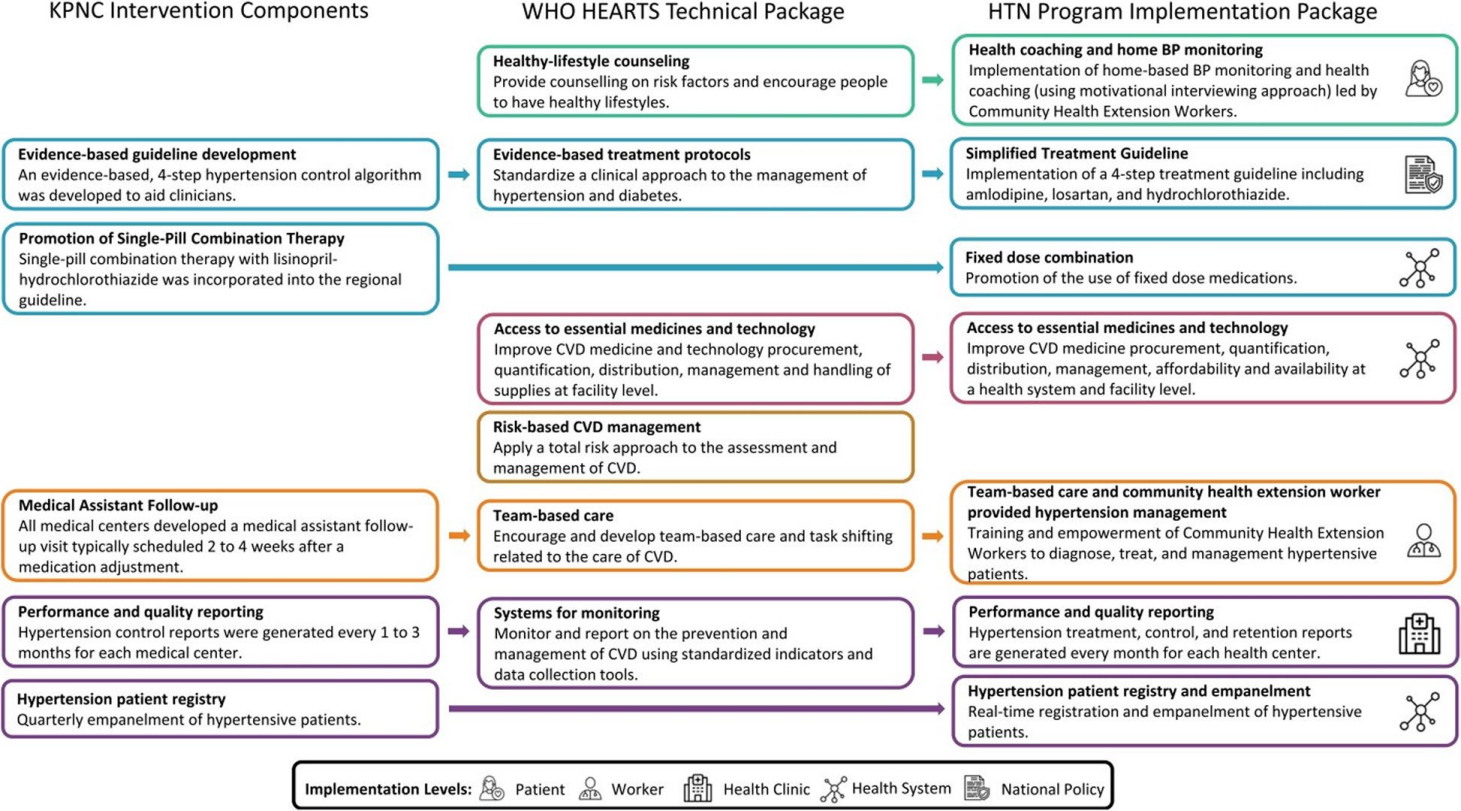
The hypertension treatment in Nigeria program implementation package. Reproduced from Baldridge et al. 2022 [6]

The Hypertension Treatment in Nigeria (HTN) Program [6] aims to address gaps in hypertension evaluation and management through the adaptation, implementation, and evaluation of the WHO HEARTS multilevel package in 60 primary healthcare centers in the Federal Capital Territory (Fig. 1). To lower hypertension-related morbidity and mortality, as well as strengthen hypertension diagnosis and management at the primary healthcare level in Nigeria, more robust primary healthcare services will alleviate time and resource burdens on both patients and healthcare providers at secondary and tertiary care centers where hypertensive services have historically been provided in Nigeria. The HTN package includes: (1) a standard treatment protocol (national policy level), (2) encouragement of fixed-dose combination therapy (health system level), (3) patient registration and empanelment (health system level), (4) incentivized team-based care (health care worker level), and (5) home blood pressure monitoring and health coaching (patient level). The HTN Program also implemented a drug-revolving fund to improve the accessibility of blood pressure-lowering medications. Results demonstrate > 90% treatment rate and > 50% hypertension control among > 21,000 registered patients from January 2020 to December 2023. If sustained and scaled up through adoption and implementation in routine public health policies and practices, the HTN Program could help reduce Nigeria’s hypertension disease burden and serve as a template for delivering integrated NCD care in primary care.

## Implementing an integrated hypertension care model – key components, early outcomes, scaling potential, and considerations

### Integrated care model with multiple reinforcing intervention components

A core strength of the HTN Program is its integrated model, incorporating multiple levels of the health system to improve hypertension treatment and control. At the national level, there is policy alignment on standard diagnosis and treatment protocols using essential medicines [7], team-based care [8], and drug revolving fund [9] implementation. Health facilities have implemented processes for continuity of care through registration and empanelment by assigning patients to care teams and tracking them longitudinally to enhance retention. By incentivizing team-based care, the HTN Program also works to strengthen relationships between patients and their local primary health centers (PHC) staff. Medication access and affordability are addressed through discounted bulk purchasing via a dedicated drug revolving fund for the 60 participating facilities. This strategy is essential in ensuring a reliable supply of quality and affordable medicines and preventing treatment interruptions, which can negatively impact blood pressure control. A key patient-level strategy includes home blood pressure monitoring and telephone-based and at-home health coaching from community health extension workers, which also represents an important approach to help people learn more about hypertension and give them more autonomy and self-efficacy in longitudinally managing their condition.

### Early successes and scalability prospects

The HTN Program’s results are promising and include a > 50% hypertension control rate across over 21,000 patients over the past 4 years. If expanded nationally, then this program could meaningfully contribute to reaching the Nigerian’s NCD targets aligned with the global 25 × 25 goal: 25% hypertension control by 2025 [10]. The high degree of local ownership and capacity building also contributes to the HTN Program’s sustainability. Most (89%) healthcare worker staff are tenured healthcare workers integrated into PHCs, reducing dependency on external partners. Systematic implementation using stepwise regional expansion can facilitate efficient scale-up for a broader population impact.

### Considerations for replication

While the Program’s initial scope focused on the Federal Capital Territory based on feasibility, the Program next seeks to demonstrate generalizability across varying socioeconomic conditions and health system capacities in 5 additional states across 5 geopolitical zones in Nigeria (i.e., Abia, Delta, Gombe, Jigawa, and Oyo). Program indicators show improvements in process metrics (e.g., number of PHC staff trained, medications dispensed) and risk factors. Recent results from a large-scale study in primary care in China have demonstrated how similar programs can improve clinical cardiovascular outcomes [11]. Assessing the impact of the HTN Program on cardiovascular events and mortality rates in Nigeria could better quantify the benefits, but this will require longer-term evaluation at a larger scale.

## Policy implications

### Leveraging the HTN program results to drive political commitment and investment in NCD care

Despite the large and rising prevalence of hypertension in Nigeria, limited priority has historically been given to NCD management, especially in primary care. The HTN Program demonstrates effective blood pressure control across 60 public primary healthcare facilities using non-physician healthcare workers with adequate supervision. These results should be championed by the Federal Ministry of Health and advocacy groups to motivate increased political attention on NCDs and allocation of public funds to better evaluate and manage NCDs within primary care.

### Facilitating the inclusion of low-cost medications from the HTN program into the national essential medicines list

Fixed-dose combination blood pressure lowering agents have been added to Nigeria’s national essential medicines list in 2023. This policy change aligns with Nigerian national treatment guidelines and the standard treatment protocol for hypertension. Efforts to expand the availability and affordability of blood pressure-lowering drugs through wider coverage under national health insurance, state health insurance, and community health insurance schemes are needed.

### Promoting implementation of the HTN care model through state primary health development agencies

Nigeria’s Primary Health Care Under One Roof initiative [12] has created institutions that are well-positioned to integrate components of the HTN Program and HEARTS package: team-based panel management leveraging community health workers, standardized treatment protocols, health information systems strengthening, and patient self-monitoring. Prioritizing hypertension and broader NCD care by state primary health care development agencies will be instrumental to long-term sustainability.

### Incorporating NCD indicators into the integrated supportive management information system

Routine monitoring of program performance metrics, like the proportion of hypertension patients with controlled blood pressure, is achievable but not yet common for frontline facilities in Nigeria. Tracking such indicators through platforms like the Integrated Supportive Supervision data system [13] would more easily enable the quality assessment of NCD care.

## Data Availability

Data will be made available through National Heart, Lung and Blood Institute (NHLBI) Biologic Specimen and Data Repository (NHLBI BioLINCC), https://biolincc.nhlbi.nih.gov/home/.

## Abbreviations

NCD: Non-communicable diseases
WHO: World Health Organization
HTN: Hypertension Treatment in Nigeria Program
PHC: Primary Health Centers
